# Complement Inhibition in the Clinic: Are We Doing Enough to Protect Patients From Infection?

**DOI:** 10.1002/eji.70233

**Published:** 2026-07-01

**Authors:** Serena Bettoni, Ebba Qviberg, Edward Chaloner, Hayley Lavender, Maisem Laabei

**Affiliations:** ^1^ School of Biochemistry and Biomedical Sciences University of Bristol Bristol UK; ^2^ Department of Life Sciences University of Bath Bath UK; ^3^ The Department of Clinical Infection, Microbiology, and Immunology Institute of Infection, Veterinary and Ecological Sciences, University of Liverpool Liverpool UK

**Keywords:** complement system, complement‐targeted therapeutics, infection risk

## Abstract

Discovered in the late 19th century as a heat‐sensitive plasma factor called “alexin”, complement was first identified for its ability to work with antibodies to destroy microorganisms. Over the past two centuries, research advances have firmly established the complement system as a fundamental component of the immune system, with broader roles in immune surveillance, inflammation, and clearing immune complexes and apoptotic debris, while also bridging innate and adaptive immunity. Due to complement playing a central role in modulating biological processes on a system‐wide scale, dysregulation or excessive activation can drive harmful inflammation and self‐tissue damage. Despite some initial safety concerns and biological complexity, therapeutic targeting of the complement system has, over the past decade, emerged as a key strategy for controlling disorders in which its unregulated activation becomes pathogenic. However, inhibition of complement, particularly at the level of C3 or C5, predisposes patients to infections, most notably by encapsulated bacteria. These include a markedly increased risk of invasive infections caused by *Neisseria meningitidis*, as well as susceptibility to *Streptococcus pneumoniae, Haemophilus influenzae*, and other opportunistic viral and fungal pathogens. In this review, we aim to describe the infection risks associated with therapeutic complement inhibition and outline emerging approaches to mitigate their complications. These include optimised vaccination protocols, antimicrobial prophylaxis, patient education, and surveillance programs, as well as next‐generation approaches such as pathway‐selective inhibitors, personalised risk stratification, and adjunctive immune support. Enhancing these protective measures will be vital to optimising the therapeutic benefit of complement inhibition while reducing infectious morbidity and mortality.

## Graphical abstract

Excessive complement activation is implicated in a broad range of diseases. Therapeutic approaches targeting the complement cascade, from pathway‐selective inhibition to terminal blockade, can effectively control disease activity. However, increasing degrees of complement inhibition are associated with a heightened susceptibility to bacterial, viral, and fungal infections, with encapsulated bacteria at the highest risk (Neisseria meningitidis, Streptococcus pneumoniae and Haemophilus influenzae). Current protection strategies include vaccination, antibiotic prophylaxis, clinical monitoring and surveillance, and patient education. Future directions aim to refine safety and efficacy through biomarker‐guided therapy, patient stratification based on genetic and clinical risk, intermittent or adaptive dosing strategies, individualised risk assessment and management, long‐term safety monitoring and strengthened ethical frameworks supported by global registry surveillance.

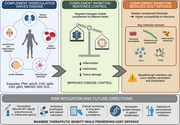

**Abbreviations**
AAV: ANCA‐associated vasculitis
aHUS: atypical haemolytic uremic syndrome
AMD: age‐related macular degeneration
ANCA: antineutrophil cytoplasmic antibody
AP: alternative pathway
AP50: 50% alternative pathway assay
C3aR: C3a receptor
C3bBbP: AP C3 convertase
C3NeF: C3 nephritic factor
C5aR: C5a receptor
CAD: cold agglutinin disease
CDC: centres for disease control and prevention
CH50: 50% classical pathway haemolytic assay
CHAPLE: CD55 deficiency with hyperactivation of complement, angiopathic thrombosis, and protein‐losing enteropathy
CP: classical pathway
CSMD: CUB and sushi multiple domains
FB: factor B
FD: factor D
FDA: Food and Drug Administration
FH: factor H
gMG: generalized myasthenia gravis
LP: lectin pathway
mAb: monoclonal antibody
MAC: membrane‐attack complex
MAPs: MBL/ficolin/CL‐associated proteins
MASP: MBL‐associated serine protease
MBL: mannose‐binding lectin
NMOSD: neuromyelitis optica system disorders
PNH: paroxysmal nocturnal haemoglobinuria
SBA: serum bactericidal activity
(Sez6): seizure‐related homolog protein 6

## 1 The Complement Cascade And Its Role In Host Defence Against Microbes

**FIGURE 1:**
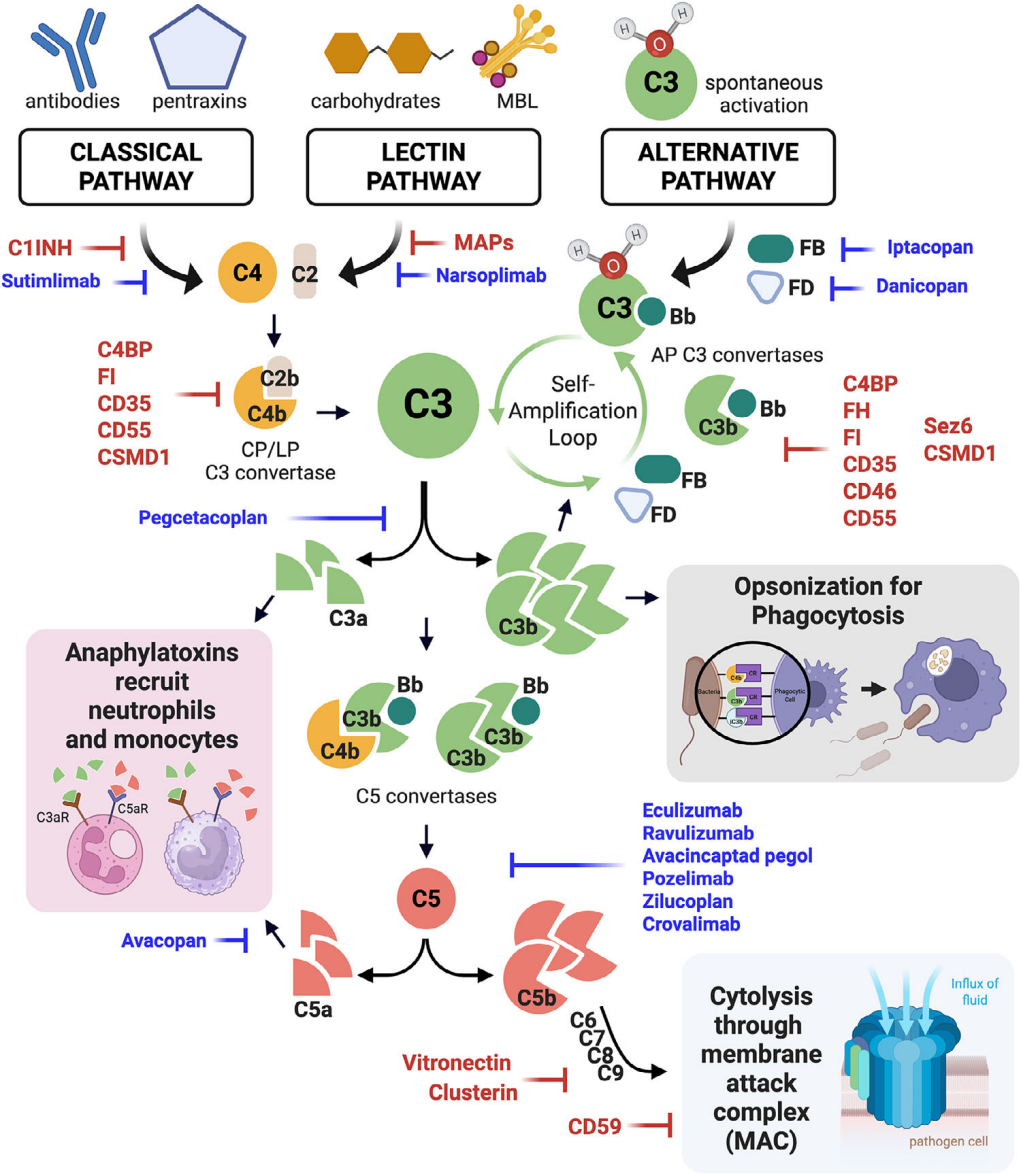
Complement system illustrating the major components, including complement regulators (red), main effector functions and currently approved complement therapeutics (blue) and their site of activity.

The complement system is arranged as a finely coordinated cascade involving over 50 components that can be activated via three separate pathways: the classical pathway (CP), triggered mainly by antibodies and pentraxins; the lectin pathway (LP), initiated by carbohydrate recognition or mannose‐binding lectin (MBL); and the alternative pathway (AP), which is continuously and spontaneously activated serving as a powerful amplification loop [1] (Figure 1). All three pathways converge at the cleavage and activation of the central complement protein C3, through C3 convertase formation, resulting in the key effector functions: opsonisation, inflammation, and cytolysis [2]. Opsonisation occurs through further cleavage and inactivation of C3b into fragments that tag pathogens for efficient uptake and destruction by binding to complement receptors CR3 and CR4 on phagocytes. Simultaneously, inflammation is triggered when anaphylatoxins C3a and C5a, small fragments released during cleavage of C3 or C5, bind to their respective receptors C3aR, C5aR1, and C5aR2 on host cells. These interactions activate signalling pathways, either proinflammatory or anti‐inflammatory, with mediators that attract immune cells to the site of infection. Cytolysis activity is achieved through the formation of a membrane attack complex (MAC) that punctures the cell membranes of unprotected pathogens, leading to their destruction.

Gram‐negative bacteria are more susceptible to direct killing by complement due to the mechanical destabilisation of the cell envelope, secondary to outer membrane disruption caused by MAC insertion [3]. In contrast, Gram‐positive bacteria and fungi are intrinsically resistant to complement‐mediated lysis due to their thick cell walls. However, complement activation on their surfaces leads to robust deposition of C3 fragments, which act as opsonins. Concurrent generation of C3a and C5a further enhances host defence by acting as potent chemotactic factors that recruit polymorphonuclear leukocytes and facilitate phagocytosis [4].

Upon activation, complement binds to nearby targets indiscriminately, including host cells in the vicinity of the triggers of complement activation. To prevent tissue damage, complement is tightly regulated by soluble and cell surface‐bound regulators [5]. Fluid‐phase inhibitors, including C1‐inhibitor (C1‐INH), MBL/ficolin/CL‐associated proteins (MAPs), factor H (FH), factor I (FI), C4b‐binding protein (C4BP), vitronectin (Vn), and clusterin (CLU), help keep continuous low‐grade fluid‐phase activation in check. Host cell membranes are protected by surface‐bound regulators, including MCP (CD46), CR1 (CD35), DAF (CD55), and CD59. Interestingly, some complement control proteins show tissue‐specific expression, such as members of the seizure‐related homolog protein 6 (Sez6) and of the CUB and sushi multiple domains (CSMD) families, which are present in the brain [6, 7]. In addition, several non‐canonical regulators of complement activation have been proposed, including the coagulation protein thrombomodulin (TM), the surface‐bound sushi domain‐containing protein 4 (SUSD4), pattern‐recognition molecules of the pentraxin family (PTXs), extracellular matrix small leucine‐rich proteoglycans (SLRPs), and others, further highlighting the complexity and adaptability of complement regulation [8].

## 2 Complement‐Mediated Disorders and Rationale for Therapeutic Complement Inhibition

Complement is vital for mounting appropriate immune responses; however, excessive complement activation or dysregulation may often be a primary driver of a wide range of inflammatory, autoimmune, neurodegenerative and thrombotic pathologies [9, 10]. Uncontrolled complement activity can arise from genetic defects in complement components or regulators, as well as from acquired factors such as persistent or excessive triggering of complement due to chronic infection, immune complexes or insufficient clearance of damaged cells or autoantibodies that stabilise activation complexes (Table 1).

**TABLE 1:** Complement‐mediated disorders, approved therapies, and infection risk.

Complement‐mediated disorders	Disease context	Approved therapies/Class/Target	Potential infection risk with treatment
Paroxysmal nocturnal haemoglobinuria (PNH)	PIGA mutation → loss of CD55/CD59 → MAC‐mediated erythrocyte lysis	Eculizumab, Ravulizumab, Crovalimab (mAb; C5) Pegcetacoplan (pegylated peptide; C3) Iptacopan (small molecule; FB) Danicopan (small molecule; FD; add‐on therapy with Eculizumab / Ravulizumab)	Known high risk for terminal (C5) or proximal (C3) inhibition: Encapsulated bacteria; *N. meningitidis*, *N. gonorrhoeae*, *S. pneumococcus*, *H. influenzae type B*. Viral infections (e.g., *herpes zoster, Cytomegalovirus, influenza*) and fungal infections (e.g., *Aspergillus, Candida*) Reduced risk of infection with FB and FD inhibition
CD55 deficiency with hyperactivation of complement, angiopathic thrombosis and protein‐losing enteropathy (CHAPLE)	Loss‐of‐function mutations in CD55 → uncontrolled complement activation → enteropathy and thrombosis	Pozelimab (mAb; C5)	Known high risk for terminal (C5) inhibition: Encapsulated bacteria; *N. meningitidis*, *S. pneumococcus*, *H. influenzae type B*
Atypical haemolytic uremic syndrome (aHUS)	Regulatory defects for loss‐of‐function mutation in CFH, CFI, CD46 or autoantibodies, or gain‐of‐function mutation in C3, CFB → alternative pathway overactivation → endothelial injury and thrombotic microangiopathy	Eculizumab (mAb; C5), Ravulizumab (mAb; C5)	Known high risk for terminal (C5) inhibition: Encapsulated bacteria; *N. meningitidis*, *N. gonorrheae*, *S. pneumococcus*, *H. influenzae type B*. Viral infections (e.g., *herpes zoster, Cytomegalovirus, influenza*) and fungal infections (e.g., *Aspergillus, Candida*)
Membranoproliferative glomerulonephritis (MPGN)	AP dysregulation (loss‐of‐function mutation in CFH, CFI, CD46 or gain‐of‐function mutation in C3, CFB) → persistent C3 activation → glomerular C3 deposition and injury	Pegcetacoplan (pegylated peptide; C3) Iptacopan (small molecule; FB)	Potential high risk proximal (C3) inhibition: Encapsulated bacteria; *N. meningitidis*, *S. pneumococcus*, *H. influenzae type B* Reduced infection risk with FB inhibition
Age‐related macular degeneration (AMD)	Genetic predisposition caused by single nucleotide polymorphisms (CFH, CFHR, CFI, C3, CFB, C2) → chronic low‐grade complement activation combined with cellular changes → retinal degeneration	Pegcetacoplan (pegylated peptide; C3) Avacincaptad pegol (pegylated RNA aptamer; C5)	Potential high risk for proximal (C3) inhibition: Encapsulated bacteria; *N. meningitidis*, *S. pneumococcus*, *H. influenzae type B*
Systemic lupus erythematosus (SLE)	Autoantibodies, immune complexes (anti‐dsDNA, anti‐Smith, anti‐Ro (SSA)/anti‐La (SSB), antiphospholipid antibodies (aPL), anti‐C1q) → classical pathway activation → systemic inflammation and tissue damage	Pegcetacoplan (pegylated peptide; C3)	Potential high risk for proximal (C3) inhibition: Encapsulated bacteria; *N. meningitidis*, *S. pneumococcus*, *H. influenzae type B*
IgA‐nephropathy (IgAN)	Galactose deficient‐IgA1 immune complexes → mesangial complement activation → glomerular injury	Iptacopan (small molecule; FB)	Reduced infection risk with FB inhibition
Anti‐neutrophil cytoplasmic autoantibody (ANCA)‐ associated vasculitis (AAV)	Antineutrophil cytoplasmic antibodies (ANCA)‐mediated neutrophil activation → C5a amplification loop → vascular inflammation	Avacopan (small molecule; C5aR)	No increased infection risk compared with standard‐care (glucocorticoid‐treated) patients; HBV reactivation reported.
Cold agglutinin disease (CAD)	IgM autoantibodies against the I antigen on the RBC → classical pathway activation → complement‐mediated haemolysis	Sutimlimab (mAb; C1s)	Potential moderate increase in infection risk from encapsulated bacteria
Generalised myasthenia gravis (gMG)	AChR autoantibodies → classical pathway activation at neuromuscular junctions → MAC‐mediated post‐synaptic damage	Eculizumab, Ravulizumab, (mAb; C5) Zilucoplan (macrocyclic peptide; C5)	Known high risk for terminal (C5) inhibition: Encapsulated bacteria; *N. meningitidis*, *S. pneumococcus*, *H. influenzae type B*
Neuromyelitis optica spectrum disorders (NMOSD)	Anti‐aquaporin‐4 autoantibodies → complement activation in central nervous system → MAC‐mediated astrocyte injury	Eculizumab, Ravulizumab, (mAb; C5)	Known high risk for terminal (C5) inhibition: Encapsulated bacteria; *N. meningitidis*, *S. pneumococcus*, *H. influenzae type B*

In conditions such as paroxysmal nocturnal haemoglobinuria (PNH) [11, 12], CD55 deficiency with hyperactivation of complement, angiopathic thrombosis, and protein‐losing enteropathy (CHAPLE) [13], atypical haemolytic uremic syndrome (aHUS) [14], membranoproliferative glomerulonephritis (MPGN) [15], and age‐related macular degeneration (AMD) [16, 17], genetic variants that increase complement activation play a primary role in the pathogenesis of the disease. PNH results from an acquired defect in glycosylphosphatidylinositol anchor synthesis, causing the loss of the surface‐bound complement regulators CD55 and CD59 from blood cells, leading to complement‐mediated haemolysis and thrombosis. The CD55 deficiency causes early‐onset protein‐losing enteropathy owing to the life‐threatening gastrointestinal disorder CHAPLE. Loss‐of‐function mutations or autoantibodies targeting regulatory proteins, such as factor H, factor I, or CD46, or gain‐of‐function mutations in AP components, including C3 or factor B (FB), trigger aHUS by inducing severe complement‐mediated injury to endothelial cells, leading to the formation of microscopic clots throughout the small blood vessels. Similar genetic defects also cause MPGN, characterised by the persistent complement activation and deposition within the glomerular basement membrane of the kidneys, leading to organ dysfunction. Specific genetic predisposition caused by single‐nucleotide polymorphisms (mainly in CFH but also in CFI, C3, CFB, and C2), leading to complement activation, combined with age‐related cellular changes in the retinal epithelium, results in the inflammatory aggregations known as drusen in AMD.

In autoimmune and inflammatory diseases, including systemic lupus erythematosus (SLE) [18], rheumatoid arthritis (RA) [19], IgA‐nephropathy (IgAN) [20], anti‐neutrophil cytoplasmic antibody (ANCA)‐associated vasculitis (AAV) [21], ischaemia‐reperfusion injury (IRI) [22], cold agglutinin disease (CAD) [23], sepsis [24] and inflammatory bowel disease (IBD) [25], complement may act as a major amplifier of disease pathology. Indeed, the presence of specific auto‐antibodies binding their antigens forms immune complexes that hyperactivate the complement cascade, exacerbating inflammation and tissue destruction in blood vessels, kidneys, joints, and lungs. Complement‐mediated tissue injury is also central to certain neurological and neuroinflammatory disorders. In generalised myasthenia gravis (gMG) and neuromyelitis optica spectrum disorders (NMOSD), pathogenic antibodies activate complement within target tissues. Complement activity has also been implicated in neurodegenerative and inflammatory conditions such as Alzheimer's disease and multiple sclerosis, although its role in these settings appears more context‐dependent [26].

Collectively, these diseases illustrate how inappropriate complement activation can harm host tissues. Growing mechanistic insights into complement‐driven disease have provided a strong rationale for therapeutic complement inhibition for many disorders.

## 3 The Expanding Therapeutic Landscape of Complement Inhibition

The humanised anti‐C5 monoclonal IgG2/4κ antibody eculizumab was the first complement therapeutic introduced into clinical practice, approved for treatment of PNH [27], validating the complement system as a pharmacological target and promoting the development of several other C5 inhibitors for clinical use (Table 1). Despite the clinical success of terminal pathway inhibition, adverse effects from ongoing activation of the proximal complement cascade, including C3‐mediated opsonisation and extravascular haemolysis, underscored the need for strategies beyond C5 blockade to control complement overactivation. The compstatin family of cyclic peptides that inhibit C3 cleavage, preventing C3b opsonisation and MAC formation, thereby limiting extravascular haemolysis, has gained importance as a therapeutic. Pegcetacoplan was approved by the FDA in 2021 for PNH [28], showing superiority over eculizumab in the phase 3 PEGASUS trial [29], in 2023 for geographic atrophy [30] and in 2025 for C3 glomerulopathy (C3G) and immune‐complex MPGN [31].

In parallel with antibody‐ and peptide‐based approaches, small‐molecule complement inhibitors have expanded the therapeutic landscape. For instance, avacopan, an orally bioavailable C5a receptor (C5aR1) antagonist, was approved in 2021 as a first‐in‐class adjunctive therapy for severe, active AAV, demonstrating efficacy and safety in the ADVOCATE trial [32].

Beyond targeting the terminal part of the cascade, specific modulation of the classical, lectin or alternative pathways has also been shown to be clinically effective. Plasma‐derived and recombinant C1‐INH products were approved in Germany in 1979 for the prophylaxis and treatment of hereditary angioedema [33]. Although this autosomal‐dominant genetic condition, caused by C1‐INH deficiency, is not strictly complement‐specific, these C1‐targeting drugs set an early precedent for proximal CP intervention. Consequently, the humanised monoclonal IgG4 antibody sutimlimab (Enjaymo), which selectively inhibits the CP protease C1s, was approved in 2022 for CAD [34], providing pathway‐specific control while preserving AP and LP activity. Oral low‐molecular‐weight inhibitors of the AP have reached the clinic, displaying complement‐inhibitory efficacy while preserving the integrity of the CP and LP. These include the FB inhibitor iptacopan (Fabhalta) [35], approved for use in PNH [36], C3G [37] and immunoglobulin A neuropathy (IgAN) [38] patients and the factor D (FD) inhibitor danicopan (Voydeya) [39], approved in 2024 as add‐on therapy to control extravascular haemolysis in PNH [40]. Likewise, to maintain the classical and alternative complement functions, essential for infection defence, the fully human monoclonal IgG4λ antibody narsoplimab (Yartemlea), which targets the effector enzyme MASP‐2 of the LP, was approved in 2025 for the treatment of transplant‐associated thrombotic microangiopathy [41].

Beyond merely validating complement as a druggable system, these advances have transitioned from inhibiting only the terminal pathway towards a diversified therapeutic arsenal that targets multiple points within the complement cascade. Ongoing efforts to achieve pathway‐selective complement modulation led to a notable rise in complement inhibitors advancing to clinical trials, including numerous first‐in‐class inhibitors targeting previously untapped nodes of the cascade, such as antibodies against C2 (empasiprubart) [42], C1q (ANX005) [43], C5a (vilobelimab) [44], properdin (tarperprumig) [45], and MASP‐3 (zaltenibart) [46]. Recent clinical trials also include innovative therapeutic approaches such as RNA interference–mediated C5 suppression (cemdisiran) [47], gene therapy using a truncated, soluble version of complement receptor 1 called mini‐CR1 (CTx001) [48], and gene therapy to regulate MAC by boosting sCD59 expression (JNJ‐1887) [49].

## 4 Increased Infection Risk in Patients Receiving Complement Inhibitor Therapies

Complement deficiencies of genetic or acquired origin are associated with increased susceptibility to infection. The risk is particularly high for infections caused by encapsulated bacteria, including *Neisseria* spp., *Streptococcus pneumoniae*, and *Haemophilus influenzae* type B [50]. Similarly, pharmacologic inhibition of the complement system confers a comparable increased risk of severe infections [51], Table 1.

Invasive meningococcal disease (IMD) caused by *Neisseria meningitidis* poses the most significant risk factor for patients undergoing terminal complement pathway inhibition. Inherited deficiencies of terminal complement components (C5–C9) are associated with a markedly increased susceptibility, 1000–10,000‐fold, to invasive meningococcal disease, frequently characterised by recurrent infections and infection with uncommon serogroups [50, 51, 52]. Clinical experience with anti‐C5 therapies has largely recapitulated this phenotype, with patients exhibiting a substantially elevated risk of meningococcal infection despite vaccination [53, 54]. Interestingly, some studies have suggested that although terminal complement deficiency increases susceptibility to infection, the resulting disease may be associated with reduced inflammatory severity and lower‐case fatality rate [55], supporting a dual role for complement in both host defence and immunopathology [51, 56].

Protection against meningococcal infection is critically dependent on complement‐mediated bactericidal activity, particularly through the formation of MAC, whereas opsonophagocytic activity plays a minimal role in host defence against IMD [56]. Moreover, the lack of capsular antibodies but the presence of high antibody titres against other surface components (e.g. lipooligosaccharide and lipoproteins) do not induce cross‐protection [57, 58]. Complement‐resistant *N. meningitidis* isolates are almost without exception encapsulated with the specific capsular polysaccharide depending on serogroup. Serogroups B and C utilise sialic acid as the chief component of the capsule, known to exacerbate FH‐mediated decay of C3b, leading to reduced C3b deposition and subsequent downstream MAC formation [59, 60, 61, 62].

Extensive evidence shows that complement inhibitors that block MAC generation increase the risk of meningococcal infection. Experimental in vitro data show significantly attenuated killing of all *N. meningitidis* serogroups in whole blood post‐eculizumab treatment [63]. Moreover, eculizumab exposure completely inhibited the killing of serogroup B even in whole blood from PNH patients previously vaccinated with the 4CMenB vaccine [64]. Non‐groupable meningococcal strains are seldom pathogenic, as found in a comparison of 667 meningococcal isolates in which non‐groupable strains represented 30%–40% of total carrier isolates, but only less than 0.2% of isolates from diseased patients [65]. However, CDC‐published adverse event (AE) data on 16 meningococcal septicaemia patients show that those treated with eculizumab are at significantly increased risk of infection with non‐groupable strains, with these representing 69% of total cases [53]. Further analysis of 239 eculizumab‐treated patient AEs from 2007–2016 highlights serogroup B meningococcal infection as the most frequently associated at 19.3% of cases, with all fatal cases involving PNH patients. Clinical trial data on 42 ravulizumab‐treated patients also show ravulizumab treatment conferring increased risk for serogroups B and Y meningococcal infections [54]. A smaller subset of reports indicated increased rates of disseminated gonococcal infection among 76 patients [66]. Other studies on AEs also suggest an increase in prevalence of gonococcal infection post‐eculizumab treatment, as well as a higher risk for infection with commensal *Neisseria* species, such as *N. cinerea*, *N. mucosa*, and *N. subflava* [67, 68]. Reports of infection by other pathogens alongside treatment of eculizumab are limited, though expectedly, the species associated are similarly encapsulated. Increases in both streptococcal and pneumococcal infections have been observed [69]. Analysis of three COMMODORE studies on 393 crovalimab‐treated and 111 eculizumab‐treated patients into risks associated with eculizumab versus crovalimab treatment reported the highest risk for pneumococcal infection, with infection rates associated with crovalimab half those of eculizumab [70].

Proximal complement inhibition blocks the entire complement cascade, inhibiting C3 activation, opsonisation and downstream terminal lysis while also interrupting C3d‐CR2 co‐stimulation, altering B‐cell activation and weakening germinal centre responses. Reports from clinical trial assessment of pegcetacoplan‐treated patients with PNH reported no meningococcal infection [71]. Adverse event data from 464 PNH patients vaccinated against *N. meningitidis*, *S. pneumoniae*, and *H. influenzae* treated with pegcetacoplan showed no encapsulated bacterial infections [71]. A case report of a PNH‐patient treated with pegcetacoplan described infection with a rare, non‐encapsulated *N. meningitidis* despite immunization with 4CMenB [72]. A case report of *H. influenzae* endophthalmitis in an 88‐year old patient undergoing intravitreal (IVT) injection of pegceptacoplan has also been described [73]. Given the importance of C3 on defence against infection, continued pegcetacoplan pharmacovigilance is essential.

Data on fungal infection risk with complement inhibitor therapy is limited; however, case reports have described *Candida dubliniensis* infection during treatment with the C5‐inhibitor ravulizumab [74]. In addition, published reports indicate that *Aspergillus* and *Candida* species account for most fungal infections observed in patients receiving eculizumab therapy [66, 75]. Recent evidence highlighting the importance of C5a driving neutrophil‐mediated fungal phagocytosis [76] and macrophage‐intrinsic C5a production sustaining mitochondrial ROS production and improved fungal killing [77] indicates potential enhanced susceptibility to fungal infections in patients treated with C3 and C5aR1 inhibitors, similar to eculizumab.

Pharmacologic inhibition of the complement system similarly confers an increased risk of viral infections. Analysis of 58,613 AE reports in the FAERS database for patients receiving C5 inhibitors (eculizumab, ravulizumab) or proximal C3 inhibitors (pegcetacoplan) highlights 11,957 reports of influenza, herpes zoster, and other viral infections, with fatal events occurring predominantly early after therapy initiation [78]. C3 inhibitors, which block the entire cascade, appear to have higher signal intensity for viral infections relative to C5 inhibitors, although overall use of C5 inhibitors accounts for most reports. Older age, female sex, and early treatment exposure are associated with increased reporting risk, while preventive vaccination or prior immunity may not fully mitigate infection risk. These findings emphasise the critical role of complement in antiviral immunity and the need for vigilance, early recognition, and potential prophylactic strategies in patients undergoing complement inhibition therapy.

## 5 Approaches to Combat Infection Risk in Patients Treated With Complement Inhibitors

### 5.1 Vaccination Strategies and Antibiotic Prophylaxis

Vaccination remains the cornerstone of prevention of infection in patients receiving complement inhibitor therapy, highlighted most notably through meningococcal vaccination, which reduces the risk of *N. meningitidis* infections in patients treated with eculizumab or ravulizumab [54]. However, vaccination alone is insufficient as an individual measure because it may not fully protect patients receiving complement therapy due to incomplete serogroup coverage, infection with non‐groupable strains [53], waning antibody titres, or impaired vaccine responsiveness for pre‐existing immunosuppressive conditions and the dependence of meningococcal clearance on terminal complement activity [79]. Indeed, meningococcal infections, including fatal cases, have been reported in vaccinated patients receiving anti‐C5 therapy [53, 54, 80]. Although vaccination contributes to protection, the degree of benefit is difficult to quantify precisely, as reduced infection rates may also reflect heightened awareness, early clinical intervention, and close monitoring of treated patients, as well as declining background incidence of meningococcal disease in many countries [82]. The persistence of meningococcal disease despite vaccination reflects the fact that vaccine‐induced antibodies normally mediate protection through complement‐dependent serum bactericidal activity (SBA). Experimental studies have demonstrated that eculizumab markedly impairs whole‐blood killing of *N. meningitidis* despite prior vaccination, highlighting that antibody responses alone cannot fully compensate for the loss of terminal complement activity [63, 64, 83]. While clearance of *N. meningitidis* is not entirely dependent on MAC‐mediated lysis, it may also involve antibody‐dependent opsonophagocytosis, Fc receptor‐mediated phagocytosis, and C3‐mediated opsonisation [64, 84]. These mechanisms appear insufficient to fully protect against invasive disease when C5 activation is blocked. This observation is consistent with the marked susceptibility of individuals with inherited terminal complement deficiencies to recurrent meningococcal infection [51, 56, 60].

Functional monitoring of vaccine response throughout treatment, including antibody titres, may refine preventive strategies. Revaccination of patients with sub‐protective titres has shown promise in mitigating the risk of meningococcal disease in a small number of patients and has potential for future successful interventions [85]. Measurement of SBA or other complement biomarkers throughout treatment may help predict infection susceptibility and guide personalised treatment selection to optimise patient outcomes [86]. In parallel, patient education remains essential. Clinicians should educate patients about the symptoms of meningococcal disease and the associated increased risk of meningococcal infections. All patients receiving complement therapeutics should seek immediate medical attention if symptoms appear, regardless of meningococcal vaccination or antimicrobial prophylaxis status, as disease progression is rapid and life‐threatening.

Given the continued occurrence of meningococcal infections despite vaccination in patients treated with complement inhibitors such as eculizumab or ravulizumab, adjunctive antibiotic prophylaxis is frequently recommended for patients receiving terminal complement inhibitors [80]. Prophylactic antibiotics, typically penicillin or an appropriate alternative in penicillin‐allergic patients, can be initiated immediately when urgent complement inhibitor therapy is required, providing additional protection during periods of high susceptibility. Long‐term antibiotic prophylaxis may be considered for the duration of complement inhibitor therapy, particularly in high‐risk patients, though evidence for optimal duration and regimen remains limited and must be balanced against the risk of resistance.

### 5.2 Selective Complement Inhibition and Degree of Pathway Blockade

Since the approval of eculizumab, therapeutic complement inhibition has evolved from broad terminal blockade towards increasingly selective, pathway‐specific, and context‐dependent strategies. While these advances have improved disease control across complement‐mediated disorders, they necessitate a refined understanding of infection risk.

Terminal pathway inhibition (C5 blockade) prevents MAC formation while preserving upstream C3 activation and opsonophagocytosis. Although this strategy reduces complement‐mediated inflammation and intravascular haemolysis, it abolishes SBA, a critical defence mechanism against encapsulated bacteria like *Neisseria* species. Consequently, patients receiving anti‐C5 therapy remain susceptible to invasive meningococcal disease [63, 64]. The alternative approach of central proximal blocking at C3 prevents amplification‐loop exacerbation but also diminishes protective roles due to early‐stage blockade. As with C5 inhibition, this method does not eliminate the risk of infection, as clinical reports of meningococcal sepsis in patients treated with C3 inhibitors have been documented [71, 72].

In vitro studies to examine the impact of new complement inhibitors provide useful information on infection risk. Studies have shown that inhibition of early complement predisposes to *H. influenzae* type b (Hib) and pneumococcus, reflecting impaired opsonisation, whereas late complement deficiency induces more susceptibility to meningococcus due to diminished MAC formation [87]. Recent therapeutic advances demonstrate that selective targeting of specific complement components can effectively control complement‐mediated diseases while maintaining certain protective immune functions against infection. Treatment with CP‐specific inhibition of C1s has also been reported with a reduced risk of bacterial infection. Clinical trial data of patients with cold agglutinin disease (CAD) treated with sutimlimab showed successful attenuation of chronic disease and improved patient quality of life, with no cases of meningococcal or other sources of infection reported [88, 89]. In vitro data reports inhibition at the level of C1s via sutimlimab preserves AP‐mediated amplification and MAC generation in the presence of pathogen‐specific anti‐capsular antibodies. Whole‐blood killing of *N. meningitidis* and *S. pneumoniae* remains largely intact when anti‐capsular antibodies are present, suggesting that selective CP inhibition may retain substantial antibacterial activity [84].

AP‐specific inhibitors provide another precision approach to hinder amplification of the cascade while enabling C3b deposition on bacteria via LP and CP activation. *In vitro* studies indicate that AP inhibitors (iptacopan and danicopan) decrease SBA to a lesser extent than anti‐C3 and anti‐C5 therapies. Indeed, the latter induces a near‐complete blockade of MAC lytic pathway activity, while oral FD and FB inhibitors achieve a more dynamic, often incomplete suppression of the cascade, thereby allowing residual complement activity through CP and LP. This partial preservation of complement function may maintain a “functional residual immunity threshold” for complement activation, which can support opsonophagocytosis and, more importantly, lytic MAC formation to clear infections. Killing of both *N. meningitidis* and *S. pneumoniae* was significantly less affected by exposure to AP‐inhibitors compared with proximal and distal inhibition; however, *H. influenzae type b* survival was improved by AP‐inhibition [90]. The apparent positive effect of AP inhibition on *H. influenzae* survival was also shown in a separate study to be vaccination status‐dependent, with levels of killing significantly less impacted in FB‐ and FD‐inhibited vaccinated samples. In the same study, C3 and C5 inhibitors significantly aided *H. influenzae type b* survival irrespective of vaccination status [87]. Taken together, AP inhibitors demonstrate an improved safety profile over C3 and C5 inhibitors in subjects with increased risk of *H. influenzae type b* infection. Moreover, comparison of the effectiveness of AP versus CP in killing *S. pneumoniae* in unvaccinated vs PCV13‐vaccinated donors showed that in unvaccinated blood, C3b‐mediated opsonophagocytosis is favoured due to relatively low antibody concentrations. However, in pre‐vaccinated blood samples, pharmacological inhibition of FB and FD did not limit bacterial killing due to increased reliance on antibody‐mediated opsonophagocytosis [91]. This data suggests that AP inhibition may increase susceptibility to invasive pneumococcal infection in non‐immune patients but will not prevent the elimination of pneumococci in vaccinated subjects. Furthermore, in vaccinated individuals, SBA against *N. meningitidis* serogroup B is abolished by inhibition of C3 or C5 but preserved when only the AP is inhibited, highlighting the critical role of the terminal complement pathway in vaccine‐mediated killing [83].

Clinical trial data for the FB inhibitor iptacopan confirmed robust AP inhibition resulting in no bacterial infections among IgAN [92] and C3G patients vaccinated against *N. meningitidis*, *S. pneumoniae*, and *H. influenzae type b* [37]. Mechanistically, complement activation via the LP, and especially the CP, by vaccine‐induced antibodies can offset AP inhibition and enable MAC formation, protecting against infection. However, robust long‐term clinical data remain limited and are needed to better define the infection risks associated with AP inhibitors.

C5a receptor antagonists, such as avacopan, represent a distinct strategy. Blocking C5a‐mediated neutrophil activation without disrupting MAC preserves terminal bactericidal function. Thus, clinical recommendations initially did not include vaccination and prophylactic antibiotics. However, reactivation of the Hepatitis B virus has been observed in patients undergoing avacopan treatment, emphasising that infection surveillance must extend beyond encapsulated bacteria [32].

Although the available evidence on infection risk from the clinic remains limited, it supports a rationale for more selective complement targeting; however, broader (non‐selective) complement inhibition may still be advantageous in acute or severe disease settings, where the immediate therapeutic benefit outweighs the comparatively lower short‐term infection risk. In contrast, for chronic or less severe conditions requiring prolonged treatment, more selective agents, such as those targeting components of the AP, may offer a safer long‐term strategy compared with non‐selective complement blockade. Importantly, with the expanding therapeutic landscape, combination therapies are emerging to tailor complement modulation rather than suppress it uniformly, balancing efficacy and host defence. For example, the oral FD inhibitor danicopan is approved as an add‐on therapy to anti‐C5 treatment for PNH, with clinical trial data indicating improved disease control through mitigation of extravascular haemolysis but without causing new infection risks [40].

### 5.3 Optimising Therapy Through Patient Stratification, Education, Dosing, and Biomarker Monitoring

Another important emerging strategy for risk mitigation during complement‐inhibitory therapy is patient stratification before treatment. Pre‐treatment screening for latent infections, such as hepatitis B and C, herpesviruses, and tuberculosis, enables appropriate management or prophylaxis before therapy is initiated. Moreover, the risk for infectious complications could be higher in young children, compared with adolescents and adults. A heightened level of vigilance should be maintained for patients with complement‐mediated kidney diseases, which constitute a large group of pathological conditions secondary to dysregulation or overactivation of the complement cascade [93]. Indeed, conditions like IgAN, C3G, IC‐MPGN, aHUS, or AAV can lead to progressive kidney damage and eventually failure, which necessitates transplantation. Their immunosuppressive medications reduce vaccine responsiveness and induce chronic inflammation [94]; therefore, these patients require vaccination and/or antibiotic prophylaxis according to an individualised schedule based on their immunosuppressive therapy.

A critical emerging consideration in the therapeutic inhibition of complement is the duration and intensity of treatment. A transient, rather than constant, blockage of the complement cascade while briefly abrogating disease defects might reduce infection risk. Indeed, short‐term or intermittently guided dosing of eculizumab for selected patients with aHUS or MPGN, coupled with monitoring of complement markers, has demonstrated disease control while potentially reducing cumulative susceptibility to infections [95, 96]. Beyond traditional complement assays assessing levels of complement components (C3, C4, C5, C2, C1q, MBL) and pathway activity (CH50, AP50), which are primarily used for diagnosis as they reflect the overall integrity or consumption of the complement cascade, a range of more sensitive activation biomarkers are increasingly being used to monitor disease activity. These biomarkers can be measured from serum, plasma, urine, whole blood, or tissue biopsies, depending on the disease context and the analyte of interest. They include soluble activation fragments, such as C3a, C5a, Bb, Ba, C3NeF, C3bBbP and sC5b‐9 [97, 98]. Cell‐bound activation products, including T cell‐bound and erythrocyte‐bound C4d, have shown utility for SLE assessment [99] while tissue complement deposits detected in renal biopsies provide valuable information in complement‐mediated kidney diseases [100]. In addition, several functional ex vivo assays have demonstrated utility in assessing complement‐targeted therapies, including endothelial cell assays evaluating deposition of C4d, C3d, and C5b‐9 to monitor aHUS progression or therapeutic response [101]. Diagnostic tests for detecting autoantibodies are also important for guiding treatment decisions and monitoring disease progression. Accordingly, a simple, fast, and cost‐effective visually based immunochromatographic test for anti‐FH antibodies has recently been described and shown to be fully adaptable for clinical use [102]. Despite the growing repertoire of complement‐targeted therapeutics and increasing research efforts to identify sensitive biomarkers, clinical implementation remains limited by technical and pre‐analytical challenges, including the need for stringent sample handling to avoid in vitro complement activation and artefactual results. Consequently, most assays are currently restricted to specialised research and reference laboratories and have not been widely implemented in routine clinical practice [103]. In parallel, multiplex immunoassays and emerging proteomic approaches [104] are improving the ability to quantify complement activity in a more mechanistic and dynamic manner, although standardisation and clinical validation remain an ongoing challenge [105].

Timing also influences vulnerability; the early phase of therapy, particularly within the first months of initiation, may represent a high‐risk window as patients adapt immunologically and vaccination responses mature. Concomitant immunosuppressive regimens potentially incorporating antibiotic prophylaxis further modulate this risk profile.

Precision strategies are outlined in the risk and mitigation guidelines (REMS) from the FDA and CDC, including biomarker‐guided dosing, vaccination monitoring, antibiotic prophylaxis, and patient stratification, all of which are essential for balancing therapeutic efficacy with the preservation of antimicrobial defence. Long‐term surveillance data will be critical to define optimal risk mitigation as increasingly proximal and selective agents enter clinical practice. Functional monitoring, including vigilance for early infection signs, remains critical, as prophylaxis does not eliminate the risk of severe disease. Patient education is therefore essential: all individuals receiving complement therapy should be instructed to seek immediate medical attention if symptoms of infection develop, regardless of vaccination or ongoing antibiotic prophylaxis, due to the rapid and potentially fatal course of the disease.

## 6 Are We Going in the Right Direction?

Complement inhibition has transformed the management of several immune‐mediated and renal diseases; however, the associated risk of infection raises important questions about whether current preventive strategies are sufficient. This risk is further amplified in nephropathy and kidney transplant patients, who are commonly treated with corticosteroids, mycophenolate mofetil, or calcineurin inhibitors. These agents suppress adaptive immunity, reduce vaccine responsiveness, and compound the immune dysfunction already present in chronic kidney disease, including impaired neutrophil and lymphocyte function, chronic inflammation, and nutritional deficiencies.

Current clinical practice relies heavily on vaccination and long‐term antibiotic prophylaxis; however, breakthrough infections still occur. Non‐adherence to prophylactic antibiotics has been documented as a contributing factor, but this alone does not fully explain the persistent burden of severe infections. This raises a central question: are we doing enough to protect these patients? While vaccination and antimicrobial prophylaxis remain essential, they may represent only the minimum standard rather than an optimal preventive approach.

Future directions should therefore move beyond simply improving existing prophylactic measures and instead challenge the current therapeutic paradigm. At present, complement is systemically inhibited, and clinicians attempt to compensate for the resulting immunological deficit with vaccines and antibiotics. A more transformative strategy would be to preserve antimicrobial defence while maintaining therapeutic efficacy. This could be achieved through tissue‐targeted complement inhibition, in which drugs act locally rather than systemically, thereby preserving circulating complement activity against pathogens [106, 107]. Similarly, pathway‐selective inhibition of AP or LP, rather than terminal complement blockade at C5, may allow disease control while sparing MAC formation and maintaining antibacterial defence.

Another promising direction is the development of biomarker‐guided or intermittent dosing strategies, in which complement suppression is adjusted dynamically according to the disease activity and relapse prediction, minimising unnecessary periods of profound immunosuppression. Improved standardisation of complement activity assays across different laboratories and expansion of the application of reliable, sensitive activation biomarkers into clinical practice would be essential for this approach, enabling clinicians to better stratify risk, monitor susceptibility, and tailor precise, individualised prophylaxis. Moreover, it is important to keep in mind that not all patients require lifelong treatment, as relapse is strongly associated with genetic background, including mutations and rare variants in CFH, CD46, and C3 [108], suggesting the importance of a complete genetic profiling for each patient.

Long‐term safety studies, head‐to‐head comparisons of complement inhibitors, and the development of improved vaccines, for instance, specifically designed for immunocompromised patients, as well as alternative prophylactic strategies, should be prioritised. Establishing international registries and global surveillance programs will also be critical to accurately quantify infection risk, identify rare complications and generate evidence that individual clinical trials cannot provide. Finally, ethical considerations surrounding lifelong complement blockade must remain central to clinical decision‐making.

Could we do more? Certainly, but the more difficult question is whether we are moving in the right direction. The goal should not simply be broader complement inhibition, but smarter and safer use of these therapies. Future progress will depend not only on developing stronger drugs, but also on balancing disease control against cumulative infectious risk and long‐term patient outcomes. Ultimately, the future should not focus solely on preventing infections caused by complement inhibition, but on designing therapies that no longer create this vulnerability in the first place.

## Author Contributions

All authors wrote and edited the manuscript.

## Conflicts of Interest

All authors declare no conflicts of interest.

## Data Availability

No new datasets were generated for this review.
